# Histological and anatomical structure of the nasal cavity of Bama minipigs

**DOI:** 10.1371/journal.pone.0173902

**Published:** 2017-03-24

**Authors:** Jingjing Yang, Lei Dai, Qinghua Yu, Qian Yang

**Affiliations:** Veterinary College, Nanjing Agricultural University, Weigang 1, Nanjing, Jiangsu, PR China; Creighton University, UNITED STATES

## Abstract

**Objective:**

The nasal mucosa is equipped with abundant lymphatic tissues, serving as the first line of defense against invasion by microorganisms. In this study, we characterized the features of the nasal mucosa of Bama minipigs (*Sus scrofa domestica*) via histological analysis.

**Methods:**

Five cross sections (I, II, III, IV, and V) were obtained from the distal end of the nasal cavity toward the pharynx (along the cavity axis) and examined. Specifically, CD3^+^ T cells, immunoglobulin A (IgA)^+^ cells, and M cells were detected by immunohistochemistry, while dendritic cells (DCs) were detected by immunofluorescence. The distribution of goblet cells was determined by periodic acid-Schiff (PAS) staining.

**Results:**

The nasal cavity of Bama minipigs can be divided into three parts: the regio vestibularis (I, II), regio respiratoria (III, IV), and regio olfactoria (V). Lymphoid tissue was present at random locations in the nasal cavity. Abundant lymphoid tissue was located in the roof of the nasopharyngeal meatus and was continuous with the lymphoid tissue of the pharynx. The distribution of CD3^+^ T cells, IgA^+^ cells, M cells, and DCs increased distally in the nasal cavity.

**Conclusions:**

The present work comprises a histological study of the nasal cavity of Bama minipigs, and will be beneficial for understanding the mechanisms of immunity in these animals after nasal vaccination.

## Introduction

The respiratory tract is the primary pathway for the infection and the spread of many diseases, including influenza and pneumonia. As the gateway to the respiratory tract, the nasal cavity plays a key role as the first line of defense against microorganism invasion. Notably, local mucosal immune responses induced by intranasal vaccine administration were shown to effectively prevent invasion and infection by pathogenic microorganisms [1–3], including by influenza viruses in humans and pigs [4, 5]. Many rodents, particularly mice, have been used in intranasal immunization research; however, experimental work carried out in mice has not translated well to human cases, the usefulness of a mouse model is limited [6–8]. Therefore, for more effective vaccine evaluation, it is necessary to use an experimental animal(s) that is more similar to humans.

Pigs have been proposed as an ideal animal model for humans because the anatomy and physiology of the porcine respiratory tract are more similar to humans than are those of rodent models [9, 10]. In particular, the sinonasal anatomy [11] and airway cell biology [12] of pigs are similar to that of humans. Indeed, minipigs were previously used in local toxicity screening of intranasally delivered drugs, as these animals could accommodate the same devices, doses, and formulations intended for human use [13]. Additionally, the pig has palatine tonsils, nasopharyngeal tonsils, and lingual tonsils within their nasal and oral cavities, which together comprise Waldeyer’s ring in humans [14]. Hence, the pig is considered an appropriate experimental animal in the field of immunology [15, 16]. Bama minipigs (*Sus scrofa domestica*) have been widely used in medical research, including in studies of laryngopharyngeal reflux [17], *Staphylococcus aureus* hepatic abscesses [18], and evaluation of drugs intended for human use [19, 20], due to their small size, high disease resistance, and ready availability. However, few minipig models of intranasal administration are available, and few studies have described the lymphoid tissues and immunocyte contents of the nasal cavity of Bama minipigs in detail. The present investigation of these tissues in Bama minipigs was motivated by the increasing interest in human applications relevant to this region. The minipig could also be used as a prospective nasal cavity model for intranasal vaccination.

## Materials and methods

### Animals

Four healthy, 2.5-month-old Bama minipigs were obtained from Jiangsu Academy of Agricultural Sciences (Nanjing, China). All animal experiments were approved by the Institutional Animal Care and Use Committee of Nanjing Agriculture University and followed National Institutes of Health guidelines for the performance of animal experiments.

### Histological analysis

**Fig 1:**
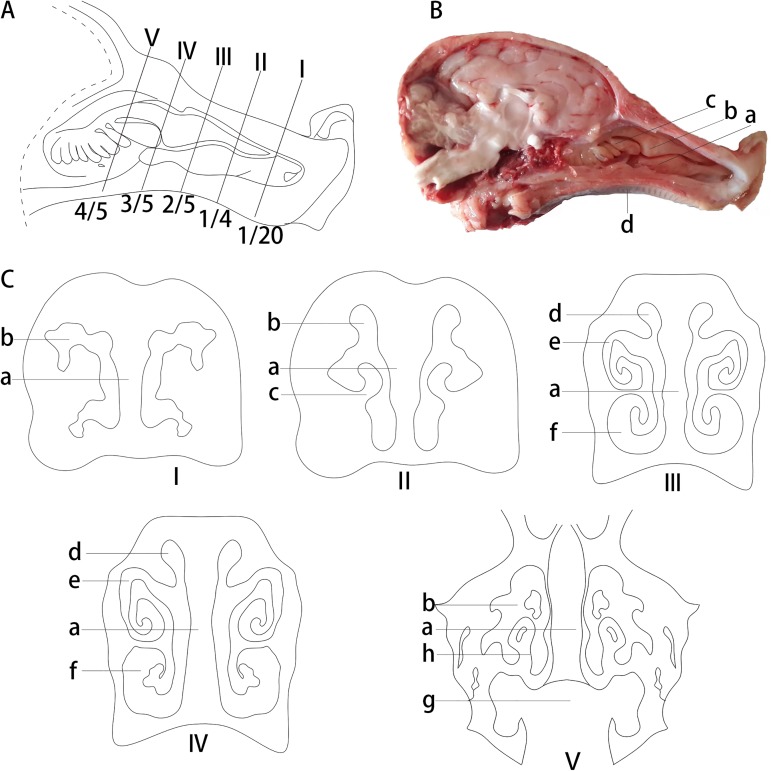
Anatomical structure of the Bama minipigs nasal cavity. (A) The position and structure of the Bama minipigs nasal cavity: (a) inferior nasal concha, (b) superior nasal concha, (c) middle nasal concha, and (d) hard palate. (B) The position of cross-sections in Bama minipigs nasal cavity. (C) Diagrams of the five cross-sections (I–V) of the Bama minipigs nasal cavity: (a) nasal septum, (b) nasal meatus, (c) inferior nasal concha, (d) superior nasal meatus, (e) middle nasal meatus, (f) inferior nasal meatus, (h) middle nasal concha, and (g) nasopharyngeal meatus.

All animals were euthanized by intravenous injection of pentobarbital sodium (100 mg/kg). After death, animals were decapitated, the lower jaw and skin were removed, and the muscles around the nasal cavity were peeled off. The nose was fixed in Bouin’s fluid for 48 hours at 20–25°C After fixation, five cross-sectional blocks were selected according to the fractions 1/20, 1/4, 2/5, 3/5 and 4/5, using the landmarks provided in the diagram presented in Fig 1A. The blocks were then decalcified by dipping into decalcifying solution consisting of 90 mL phosphate-buffered saline (PBS), 5 mL 4% paraformaldehyde solution, and 5 mL formic acid for 1 week. Before processing, the blocks were divided equally into right and left parts using a scalpel. Blocks from parts of the nose (paranasal siuns and lacrimal duct) were cut to fit the slides and dehydrated by treatment with a graded alcohol series (75%, 85%, 95%, 100%, 100% ethanol). The dehydrated blocks were then embedded in paraffin, serially sliced into 6-μm-thick sections, and mounted on slides. The sections were dried horizontally on a warming tray overnight at 37°C. Cross-sectional sections (I–V) were stained with hematoxylin-eosin (HE) or periodic acid-Schiff (PAS), or subjected to immunohistochemical (IHC) or immunofluorescence analyses, respectively. Integral images were scanned using a BX51 Digital Camera System (Olympus Inc., Tokyo, Japan).

### Immunohistochemical detection of CD3^+^ T cells, immunoglobulin A (IgA) ^+^ cells, and M cells

After deparaffinization and rehydration, paraffin sections were rinsed in PBS and incubated at 90–95°C for 30 min in citrate buffer (pH 6) for antigen demasking. Then, sections were treated for 10 min with 0.3% H_2_O_2_ in PBS to quench endogenous peroxidase, treated with streptavidin-biotin complex (SABC) (BOSTER, Wuhan, Hubei, China), and washed in PBS. After blocking with 5% normal goat serum or 5% bovine serum albumin, sections were incubated with rabbit anti-pig CD3, goat anti-pig IgA, and mouse anti-human cytokeratin 18 antibodies (Table 1) overnight at 4°C, and for 30 min at room temperature, in a humidified chamber. Stained sections were then washed in PBS, incubated with biotinylated secondary antibodies (Table 1) for 60 min, and treated with SABC for 60 min. Positive cells were visualized by treatment with diaminobenzidine. The respective isotype controls were used as negative controls. Sections were counterstained with hematoxylin and images were obtained using a light microscope (BH-2; Olympus).

**Table 1:** The information of antibodies.

	Name	Dilution	Company	City
Primary antibody	Rabbit anti-pig CD3 antibody	1:500	Abcam	Hong Kong
	Mouse anti-human Cytokeratin 18 antibody	1:1200	Abcam	Hong Kong
	Goat anti-pig IgA antibody	1:200	Bethyl	Montgomery
	FITC-conjucated mouse anti-pig monocyte+granulocyte (SWC3a) antibody	1:200	Abcam	Hong Kong
	PE-conjucated mouse anti-pig HLA-DP/DR (MHCII) antibody	1:200	LSBio	Seattle
Secondary antibody	SABC-POD (rabbit IgG) Kit		BOSTER	Wuhan
	SABC-POD (mouse IgG) Kit		BOSTER	Wuhan
	SABC-POD (goat IgG) Kit		BOSTER	Wuhan

### Immunofluorescence (IF) staining of dendritic cells (DCs)

For IF staining of DCs, tissue sections were rinsed and subjected to antigen demasking as described above. After rinsing in PBS, sections were treated with 5% normal goat serum for 20 min, incubated with fluorescein isothiocyanate (FITC)-conjugated mouse anti-pig monocyte-granulocyte (Abcam, Hong Kong, China) and phycoerythrin (PE)-conjugated mouse anti-pig HLA-DP/DR (LSBio, Seattle, WA, USA) antibodies (Table 1) overnight at 4°C in a moist chamber. PBS was used in place of the anti-pig antibody for the control. After rinsing in PBS, sections were counterstained with 4′,6-diamidino-2-phenylindole (DAPI) for 5 min, and observed under a confocal laser microscope (LSM-710; Zeiss, Oberkochen, Germany).

### Statistical analysis

Immunocyte numbers were calculated using Image Pro software (ipwin32; Media Cybernetics, Silver Spring, MD, USA). Positive cells were counted randomly in 10 fields per slice (n1 = 3) from each cross section from Bama minipigs (n2 = 4). Statistical analysis was performed using SPSS software (ver. 17. 0; SPSS Statics, Inc., Chicago, IL, USA). Statistical significance was determined by one-way analysis of variance (ANOVA) followed by Duncan’s test. *P* values less than 0.05 were considered statistically significant.

## Results

### Anatomical characteristics of the nasal cavity

The mean nasal cavity length of the Bama minipigs tested, measured from the tip of the nose to the nasopharynx, was 8.5 ± 0.42 cm. Five cross-sections (I, II, III, IV, and V) taken at fractions 1/20, 1/4, 2/5, 3/5, and 4/5 were subsequently selected for examination of the nasal cavity structure; the cavity is divided into right and left sides by the cartilaginous nasal septum and consists of the superior nasal concha, inferior nasal concha, and middle nasal concha (Fig 1B). The nasal cavity was partitioned into three regions: the regio vestibularis (I, II), regio respiratoria (III, IV), and regio olfactoria (V). The superior nasal concha was long, the inferior nasal concha was short and wide, and the middle nasal concha was small and located ventral to the superior nasal concha. The concha nasalis, located in the middle of the nasal cavity, was spiral-shaped, and the nasopharyngeal meatus was found to be contiguous with the pharynx towards the rear of the nasal cavity.

### Histological characteristics of the nasal cavity

**Fig 2:**
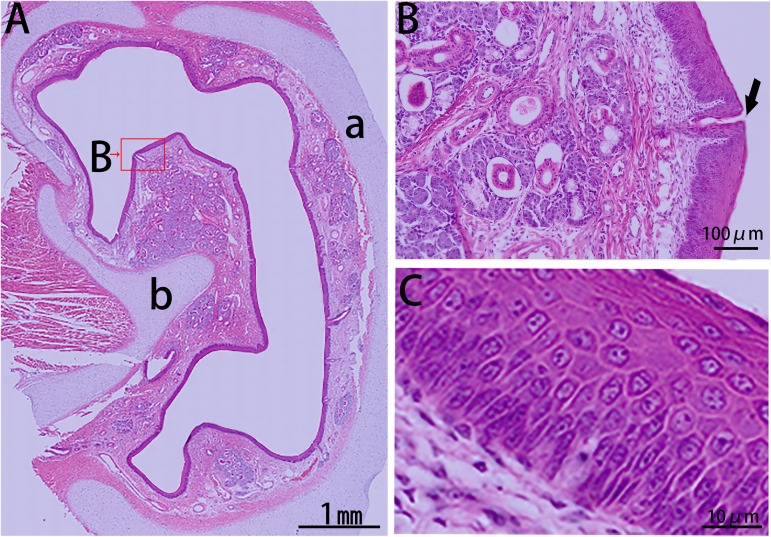
HE stain of csI from the right side of the Bama minipigs nasal cavity. (A) Panoramic scanning of csI: (a) nasal septum and (b) inferior nasal concha. (B) Blood vessels and glands located in the lamina propria. Arrow indicates the opening of the duct to the nasal cavity. (C) Stratified squamous epithelium.

**Fig 3:**
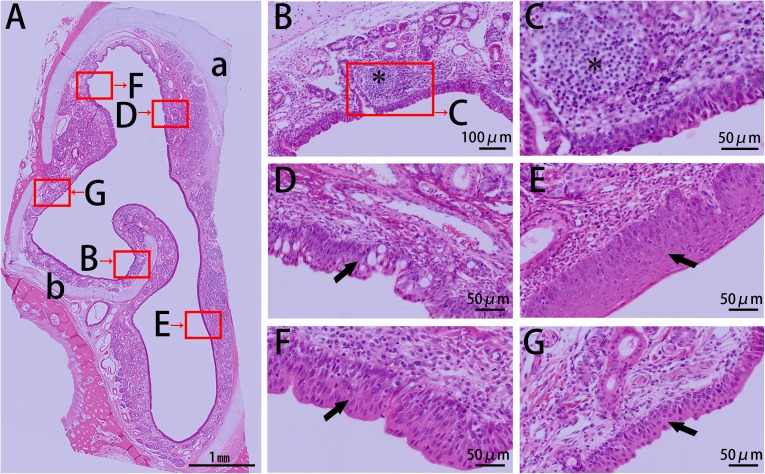
HE stain of csII from the right side of the Bama minipigs nasal cavity. (A) Panoramic scanning of csII: (a) nasal septum and (b) inferior nasal concha. (B, C) Lymphoid follicle under the mucosal epithelium on the dorsal side of the inferior nasal concha (asterisk). (D–G) Transition from stratified squamous epithelium to pseudostratified columnar ciliated epithelium: (D) columnar epithelium consisting of goblet cells, (E) thick stratified squamous epithelium, (F) columnar epithelium, and (G) thin stratified squamous epithelium.

The first block (cross section I, csI) of the nasal cavity was covered entirely by stratified squamous epithelia (Fig 2), and capillary vessels and glands were distributed abundantly in the lamina propria. While csII was mostly covered with stratified squamous epithelia, this section exhibited a transition towards a pseudostratified columnar ciliated epithelium (Fig 3). The non-ciliated cuboidal or columnar cells, basal cells, and goblet cells of this tissue were observed at the dorsal nasal meatus, and lymphoid follicles were primarily located on the dorsal side of the inferior nasal concha.

**Fig 4:**
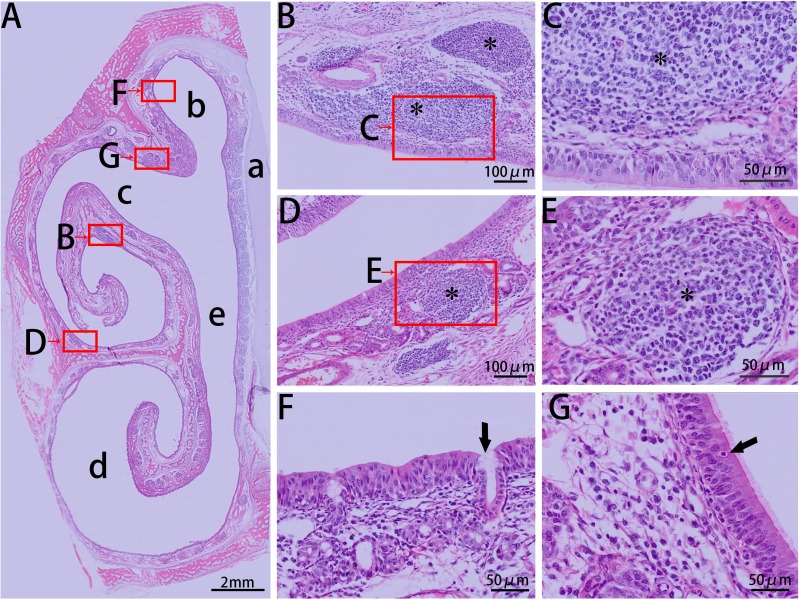
HE stain of csIII from the right side of the Bama minipigs nasal cavity. (A) Panoramic scanning of csIII: (a) nasal septum, (b) superior nasal meatus, (c) middle nasal meatus, (d) inferior nasal meatus, and (e) common nasal meatus. (B, C) Lymphatic tissue located on the dorsal medial inferior nasal concha (asterisk). (D, E) Lymphoid follicle under the mucosal epithelium of the dorsal inferior nasal concha (asterisk). (F) Columnar epithelium and mucous glands (arrow) on the surface of the superior nasal concha. (G) Pseudostratified columnar ciliated epithelium and intraepithelial lymphocytes (arrow).

In csIII, the stratified squamous epithelium had predominantly transitioned to pseudostratified columnar ciliated epithelia, except for the lateral wall of the superior nasal meatus which was lined by non-ciliated columnar epithelium. There was no clear boundary between pseudostratified columnar ciliated epithelia and non-ciliated columnar epithelia. More mucous glands and capillary vessels were observed in the lamina propria of the mucosa of the dorsolateral spiral of the inferior nasal concha, and the mucosa appeared to be much thicker than that in the ventromedial spiral of the inferior nasal concha. Lymphoid follicles were observed in this region, located on the ventromedial side of the dorsal spiral of the inferior nasal concha and the lateral side of the nasal cavity (Fig 4).

**Fig 5:**
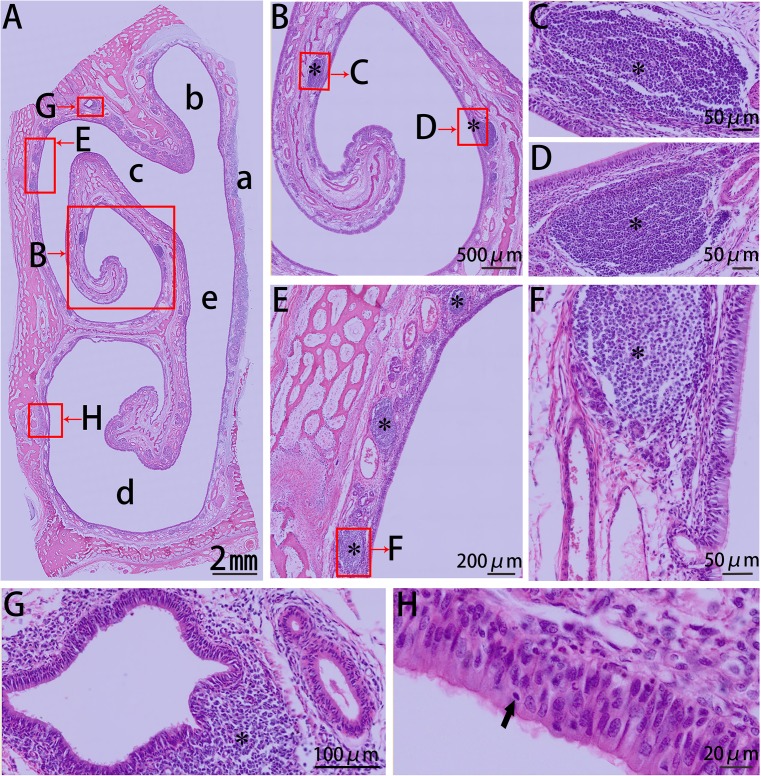
HE stain of csIV from the right side of the Bama minipigs nasal cavity. (A) Panoramic scanning of csIV: (a) nasal septum, (b) superior nasal meatus, (c) middle nasal meatus, (d) inferior nasal meatus, and (e) common nasal meatus. (B–D) Lymphoid follicle under the mucosal epithelium of the dorsal medial inferior nasal concha (asterisk), FAE consisted of pseudostratified columnar ciliated epithelium and simple epithelium. (E, F) Lymphoid follicle under the mucosal epithelium of the dorsolateral nasal cavity. (G) Lymphatic tissue (asterisk) located on the dorsal nasal cavity near the lumens lined by respiratory epithelium. (H) Pseudostratified columnar ciliated epithelium and intraepithelial lymphocytes (arrow).

CsIV was covered entirely by pseudostratified columnar ciliated epithelia (Fig 5), and there was a thick dorsolateral spiral of the inferior nasal concha. Lymphatic tissues were found mainly on the lateral side of the nasal cavity and on the ventromedial side of the dorsal spiral of the inferior nasal concha. Additionally, the epithelium of the nasopharynx-associated lymphoid tissue (NALT) in these regions was often transformed into follicle-associated epithelium (FAE) by infiltrating lymphoid cells, M cells, and DCs.

**Fig 6:**
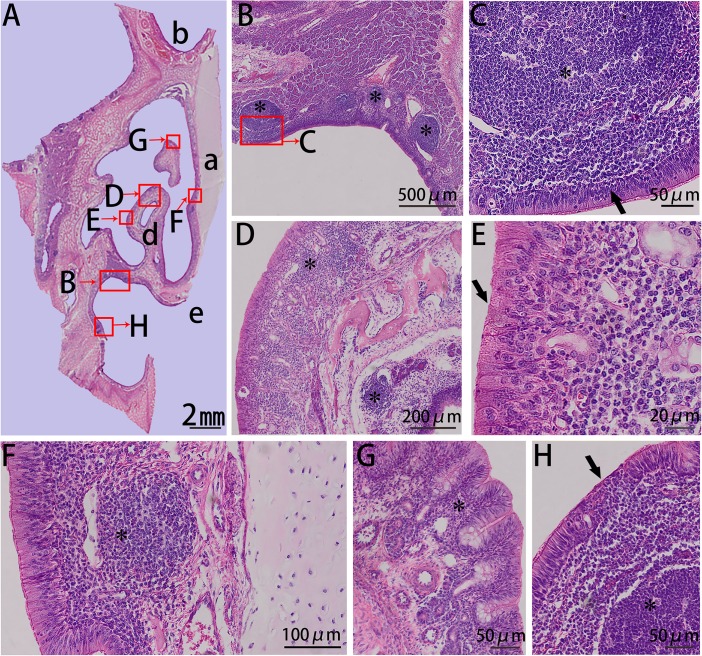
HE stain of csV from the right side of the Bama minipigs nasal cavity. (A) Panoramic scanning of csV: (a) nasal septum, (b) frontal sinus, and (c) nasopharyngeal meatus. (B) Aggregation of lymphocytes located on the roof of the nasopharyngeal meatus (asterisk). (C) Lymphatic tissue (asterisk) located on the roof of the nasopharyngeal meatus and the FAE (arrow) consisting of ciliated cells. (D) Lymphoid follicles (asterisk) distributed under the mucosal epithelium of the middle nasal concha. (E) Columnar epithelium (arrow). (F) Lymphatic tissue (asterisk) located on the wall of the nasal cavity and under the columnar epithelium of the nasal septum. (G) Mucous glands located on the dorsal side of the middle nasal concha and lymphoid tissue (asterisk) distributed randomly between or under the mucous glands. (H) Lymphatic tissue located on the lateral side of the nasopharyngeal meatus (asterisk) and lymphoepithelium (arrow).

Lastly, csV harbored no superior nasal concha or inferior nasal concha, and only slight amounts of middle nasal concha and nasopharyngeal meatus (Fig 6). The covering epithelium was olfactory epithelium, and the lymphatic tissues in this region were primarily on the dorsal side of the middle nasal concha and on the roof of the nasopharyngeal meatus, which was contiguous with the lymphoid tissue of the pharynx (termed the pharyngeal tonsils).

### Immunocyte characteristics of the nasal cavity

**Fig 7:**
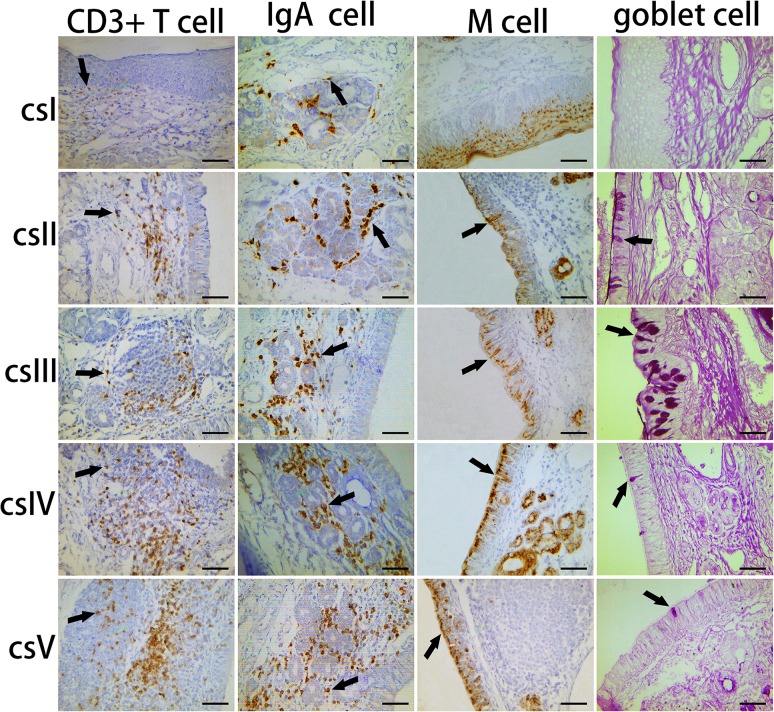
The distribution pattern of CD3^+^ T cells, IgA^+^ cells, M cells, and goblet cells in the Bama minipigs nasal cavity. CD3^+^ T cells, IgA^+^ cells, and M cells examined using IHC (arrows indicate positive cells). Goblet cells in the surface epithelium are shown by AB/PAS staining (arrows indicate positive cells). Scale bar = 100 μm.

**Fig 8:**
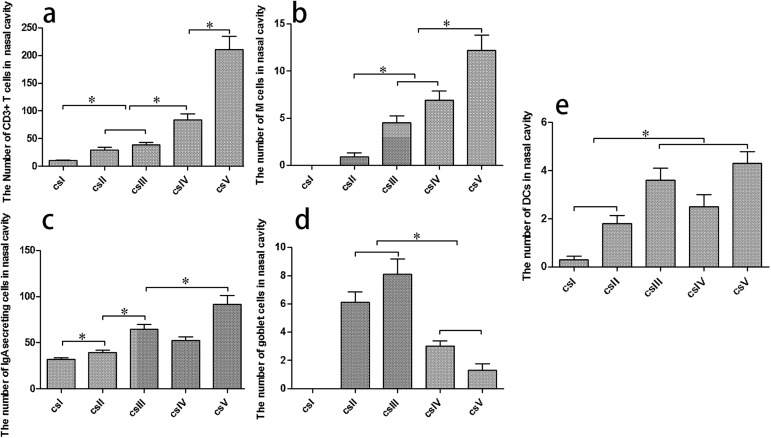
Quantitative analysis of immunocytes in the Bama minipigs nasal cavity. The number of immunocytes was counted from five cross sections in a unit area (40×). (a) CD3^+^ T cells, (b) M cells, (c) IgA^+^ cells, (d) goblet cells, and (e) MHC II^+^SWC3a^+^DCs. *P < 0.05.

M cells and goblet cells function as entry sites for many antigens. IHC analysis using antibodies specific to cytokeratin 18, a marker of porcine M cells [21], demonstrated that M cells were located in the epithelium of Bama minipigs, where they exhibited features typical of such cells, namely a columnar structure that is primarily interspersed throughout the FAE (Fig 7). In contrast, there was no typical cell morphology in PBS (S1C Fig) and isotype control (S1F Fig).The distribution pattern of M cells indicated an increase from the proximal to distal side in the nasal cavity (Fig 8). Meanwhile, goblet cells were detected in the surface epithelia of the nasal mucosa of Bama minipigs by PAS staining (Fig 7). These cells, which appeared blue after staining, were typically circular and cup-shaped and were located in the middle nasal cavity (Fig 8).

**Fig 9:**
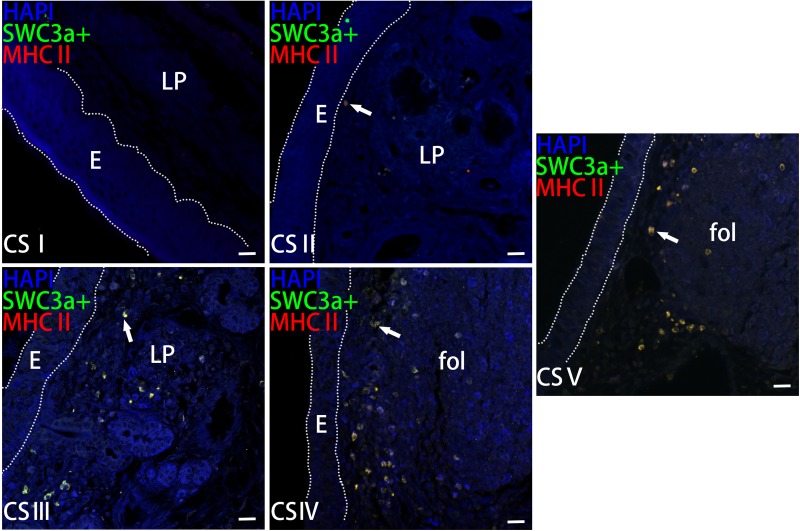
The localization of MHCII^+^SWC3a^+^ DCs in the Bama minipigs nasal cavity. Paraffin sections of the nasal cavity were stained with SWC3a (green), MHCII (red), and 4’,6-diamidino-2-phenylindole (DAPI; blue). DCs are indicated by arrows. E: epithelium, LP: lamina propria, fol: lymphoid follicle, scale bar = 20 μm.

DCs were detected by IF analysis via dual staining with antibodies specific to the DC markers major histocompatibility complex II (MHCII) and SWC3a (Fig 9). MHCII^+^SWC3a^+^ DCs were distributed around lymphoid follicles, but were also abundant in the lamina propria, which is the loose connective tissue underlying the epithelium. DCs were predominantly located in the rear of the nasal cavity, in which many lymphoid tissues were also distributed (Fig 8).

Lastly, the distribution patterns of CD3^+^ T cells and IgA^+^ in the nasal cavity of Bama minipigs were examined by IHC staining using appropriate antibodies (Fig 7). Compared with PBS (S1A and S1B Fig) and isotype control (S1D and S1E Fig), cell membranes were stained dark brown. In the lamina propria, CD3^+^ T cells appeared oval or circular, particularly in the periphery of lymphoid follicles, and their distribution increased from the proximal to distal side of the nasal cavity (Fig 8). The surface of IgA^+^ cells showed the presence of SIgA molecular adhesion. Similar to CD3^+^ T cells, these cells were also oval or circular in the lamina propria, particularly around the glands, and were predominately located in the rear of the nasal cavity (Fig 8).

## Discussion

In this study, we examined five cross sections of the nasal cavities of Bama minipigs; section selection was based on the presence of the main nasal structures and of distinct representative epithelial areas, although the landmarks used differed from those reported for Göttingen minipigs [22]. Our approach was geared toward observation of the general histological and anatomical structures of the nasal cavity of Bama minipigs. Conversely, the accessory nasal structures of these animals, such as the lacrimal ducts and paranasal sinus, fell outside of the tissue blocks used and were therefore not examined. The distribution of nasal epithelial populations differs among species [23]. In addition to the four principal nasal epithelial tissues (squamous, transitional, olfactory, and respiratory tissues) the lymphoepithelium, which overlies the NALT, was located in the floor at the opening of the nasopharyngeal meatus. The lymphoepithelium in rodents is restricted to the ventral aspects of the lateral walls at the opening of the nasopharyngeal duct [24]. Similar to nasal epithelium, the lymphoepithelium of adenoids has well-developed tight junctions that play an important role in the barrier function [25].

Due to the proximity to the FAE that lines the nasal epithelium, NALT contributes to process antigens, thereby inducing the mucosal immune response. The NALT in rodents consists of pairs of lymphoid tissues located in the floor of the nasal cavity [26]. We observed that, in the noses of Bama minipigs, the NALT is located adjacent to the nasopharyngeal meatus roof, and is continuous with pharynx lymphoid tissue, similar to the nasopharyngeal tonsils of Göttingen minipigs and humans [22]. Lymphoid follicles, as part of the NALT, were accumulated in random areas within the nasal mucosa of the Bama minipigs tested. The presence and size of lymphoid follicles depends on species, age, and exogenous stimuli [27, 28]. The follicles are diffuse in the human nose [29], as well as in the noses of rabbits and ducks [30, 31].

In addition to their primary functions of producing and secreting mucus, goblet cells can be central to mucosal immunity. Goblet cell-derived resistin-like molecule-β plays a critical role in recruiting CD4^+^ T cells [32]. In addition, small intestinal goblet cells have been shown to acquire luminal antigens and present to CD103^+^DCs in the lamina propria [33]. In this study, a few goblet cells were observed between the epithelium of the Bama minipig nasal cavity, similar to the distribution of goblet cells seen in humans [34]. M cells comprise another important immune cell in the epithelium. In Bama minipigs, these cells were primarily situated in the rear part of the FAE, where lymphocytes also aggregate. Respiratory M cells play a critical role as a gateway to the upper airway [35], and have been shown to serve as entry portals for antigens in human nasopharyngeal lymphoid tissue [36], including influenza A virus antigens [37]. M cells are situated directly adjacent to DCs in the dome areas of intestinal Peyer’s patches [38]. DCs are dedicated antigen-presenting cells that serve as a surveillance system to sense infections [39]. In humans, DCs are often located in the tissues that line the airways as opposed to blood [40]. Our results show that, in Bama minipigs, MHCII^+^SWC3a^+^ DCs mainly accumulated in the rear of the nasal cavity. DCs deliver antigens to nearby T/B cells and thus induce an immune response. In our minipigs, many NALT CD3^+^ T cells and IgA cells were located in the rear of the nasal cavity. Therefore, the principal role of these cells appears to be the induction of a strong nasal mucosal immune response.

Even with improvements in modern medicine, infectious respiratory diseases remain an important threat to human health. Intranasal immunization is a non-invasive and easily administered treatment that allows for rapid absorption of antigens, thereby protecting against respiratory infections [41]. In addition, although both intranasal and oral administration elicit immune responses, the intranasal delivery route produces a superior immune effect [42]. Some studies have highlighted the safety and efficacy of intranasal immunizations for influenza [43]. Future studies may focus on the nasal cavity of experimental animals used in toxicology research. The present work comprises a histological study of the nasal cavity of Bama minipigs that can serve as a reference point for future analyses of the immune mechanisms elicited by nasal vaccination.

## Supporting information

S1 Fig The control of the immunohistochemistry. We performed immunohistochemical staining to show CD3^+^ T cells, IgA^+^ cells and M cells. We used the isotype antibody and phosphate-buffered saline (PBS) instead of primary antibody as negative controls. (A-C) PBS was used as anti-CD3 (A), pig IgA (B) and anti-cytokeratin 18 (C) negative controls. (D-F) Three kinds of isotype antibodies were used as anti-CD3 (D), pig IgA (E) and anti-cytokeratin 18 (F) negative controls respectively. Scale bar = 100 μm.
(TIF)
