# Supplementary figures and images for: Histological and anatomical structure of the nasal cavity of Bama minipigs
Source: PLoS One. 2017 Mar 24;12(3):e0173902. doi: 10.1371/journal.pone.0173902 (PMC5365122; doi:10.1371/journal.pone.0173902)

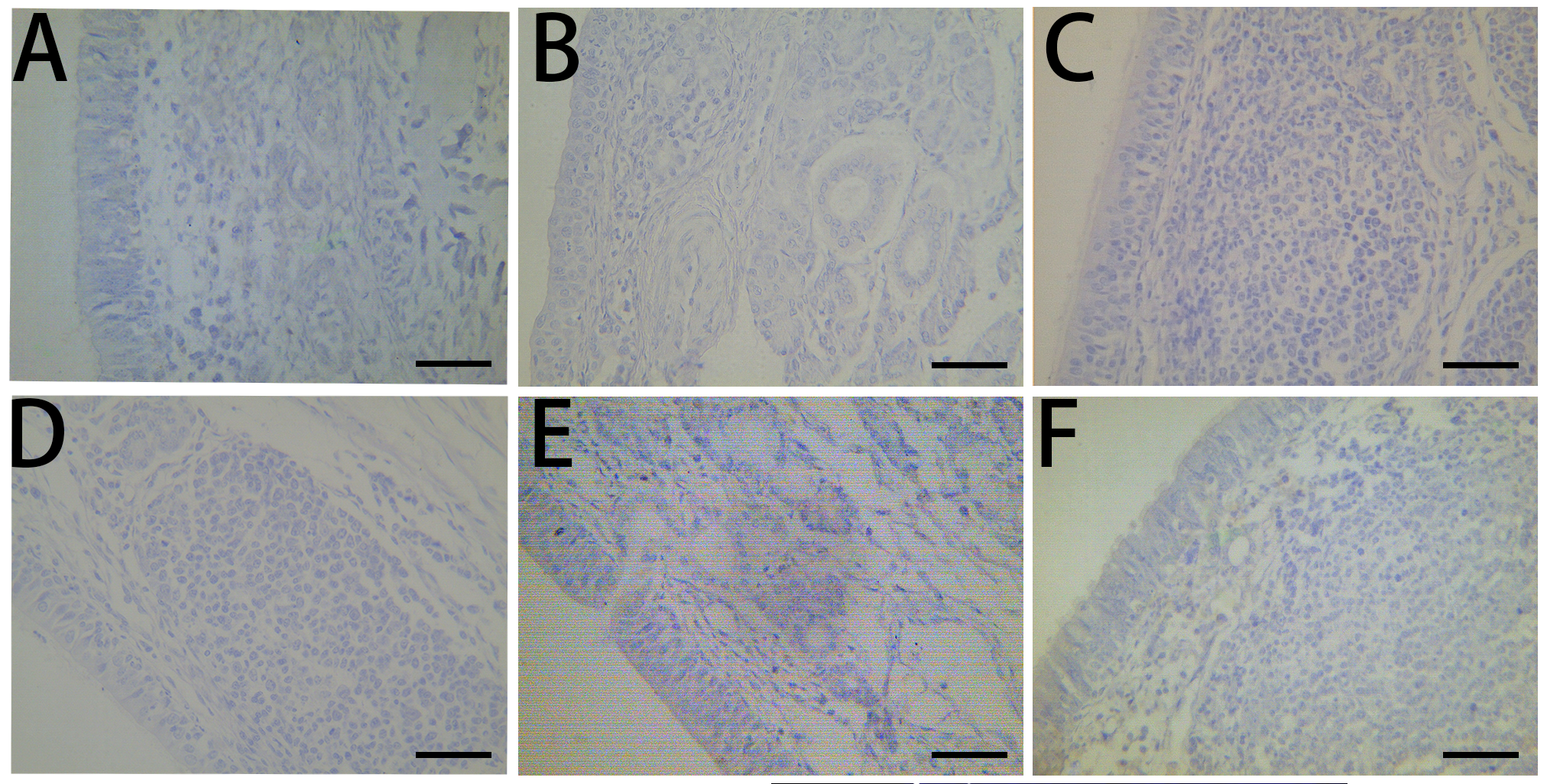

Supplement: S1 Fig — We performed immunohistochemical staining to show CD3+ T cells, IgA+ cells and M cells. We used the isotype antibody and phosphate-buffered saline (PBS) instead of primary antibody as negative controls. (A-C) PBS was used as anti-CD3 (A), pig IgA (B) and anti-cytokeratin 18 (C) negative controls. (D-F) Three kinds of isotype antibodies were used as anti-CD3 (D), pig IgA (E) and anti-cytokeratin 18 (F) negative controls respectively. Scale bar = 100 μm. (TIF) [file pone.0173902.s001.tif]
